# Antibiotic Resistance: One Health One World Outlook

**DOI:** 10.3389/fcimb.2021.771510

**Published:** 2021-11-25

**Authors:** Bilal Aslam, Mohsin Khurshid, Muhammad Imran Arshad, Saima Muzammil, Maria Rasool, Nafeesa Yasmeen, Taif Shah, Tamoor Hamid Chaudhry, Muhammad Hidayat Rasool, Aqsa Shahid, Xia Xueshan, Zulqarnain Baloch

**Affiliations:** ^1^ Department of Microbiology, Government College University Faisalabad, Faisalabad, Pakistan; ^2^ Institute of Microbiology, University of Agriculture, Faisalabad, Pakistan; ^3^ College of Veterinary Medicine, South China Agricultural University, Guangzhou, China; ^4^ Faculty of Life Science and Technology, Kunming University Science and Technology, Kunming, Yunnan, China; ^5^ Public Health Laboratories Division, National Institute of Health, Islamabad, Pakistan; ^6^ Faculty of Rehabilitation and Allied Health Sciences, Riphah International University, Faisalabad, Pakistan

**Keywords:** One Health, antibiotic resistance, human, animal, environment

## Abstract

Antibiotic resistance (ABR) is a growing public health concern worldwide, and it is now regarded as a critical One Health issue. One Health’s interconnected domains contribute to the emergence, evolution, and spread of antibiotic-resistant microorganisms on a local and global scale, which is a significant risk factor for global health. The persistence and spread of resistant microbial species, and the association of determinants at the human-animal-environment interface can alter microbial genomes, resulting in resistant superbugs in various niches. ABR is motivated by a well-established link between three domains: human, animal, and environmental health. As a result, addressing ABR through the One Health approach makes sense. Several countries have implemented national action plans based on the One Health approach to combat antibiotic-resistant microbes, following the Tripartite’s Commitment Food and Agriculture Organization (FAO)-World Organization for Animal Health (OIE)-World Health Organization (WHO) guidelines. The ABR has been identified as a global health concern, and efforts are being made to mitigate this global health threat. To summarize, global interdisciplinary and unified approaches based on One Health principles are required to limit the ABR dissemination cycle, raise awareness and education about antibiotic use, and promote policy, advocacy, and antimicrobial stewardship.

## Introduction

Antibiotic resistance (ABR) is a global health concern that has been linked to humans, animals, and environmental factors. ABR necessitates a multidisciplinary, multisector, and coordinated approach to address health threats at the human-animal-environment interface, which are covered under the umbrella of the One Health concept (Robinson et al., 2016). One Health recognizes the inextricable link between humans, animals, and the environment to achieve better community health and well-being. One Health is an interdisciplinary and holistic concept considering the interdependent human and animal health in association with the ecosystem, where they live. The leading regulatory authorities such as the International Monetary Fund (IMF), the World Bank, the World Health Organization (WHO), and the G8 declared ABR as a major global health threat of the 21^st^ century. All these forums affirmed that ABR needs coordinated and interdisciplinary efforts because different ecosystems participate in the acquisition, emergence, and distribution of ABR (Hernando-Amado et al., 2019). The emergence of ABR and transmission dynamics of multi-drug resistant pathogens comes under One Health case studies suggesting an indispensable collaborative role of human, animal, and environmental professionals in mitigation of global ABR.

In a global context, the “One Health One World” concept integrates molecular epidemiological aspects that add to understanding the evolution or genetic relatedness of ABR in pathogens/vectors, host (human/animal), and the associated environment on a global scale. The socioeconomic factors such as world trade, conflict, displacement, travel, human, and animal migration are important drivers of the global dissemination of ABR (McMichael, 2015; Hernando-Amado et al., 2019). Whereas, locally, it emphasizes geographically close ecosystems, which play a crucial role in the emergence and distribution of ABR. Recently, in this scenario, the Chile-Sweden collaboration has taken the One Health– One World initiative to control ABR at a global level (Cabrera-Pardo et al., 2019).

**Figure 1:**
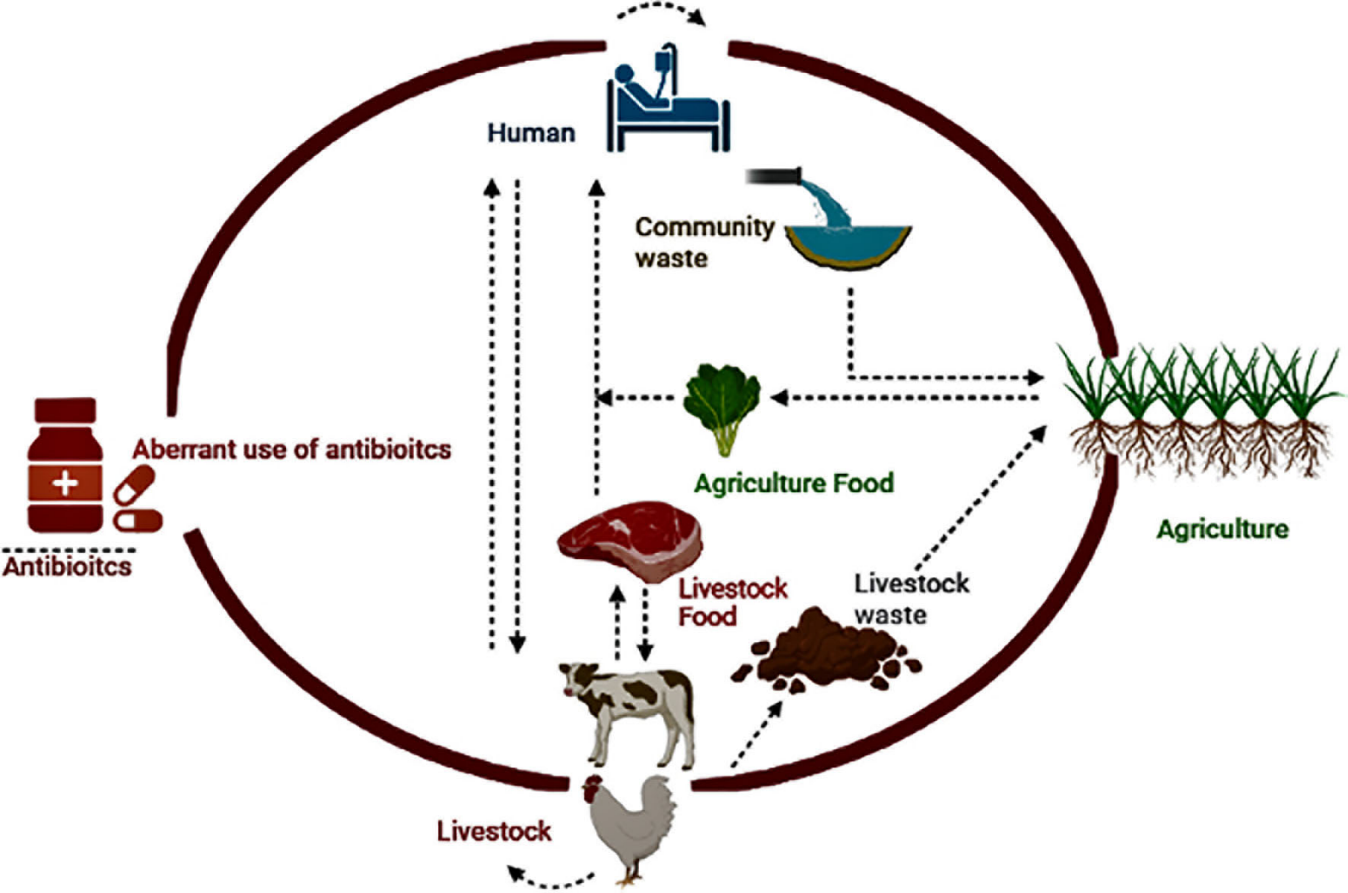
Potential One Health drivers associated with ABR.

Inadequate antibiotic use in animals and humans, contaminated environments, and ineffective infection control policies are among the causes of ABR’s local and global spread (Marti et al., 2014; Burow and Käsbohrer, 2017). Resistance reservoirs have emerged due to the irrational use of antibiotics in humans, animals, communities, and associated environments, resulting in the persistence of drug residues or resistance genes in the environment. Multiple environmental reservoirs are part of ABR dissemination, including soil, water, hospital, industrial, farm waste, and various polluted ecological niches (Marti et al., 2014; Huijbers et al., 2015) (**Figure 1**). The drivers of the local and global distribution of ABR include imprudent use of antibiotics in animals and humans, contaminated environment, and inadequate infection control policies (Marti et al., 2014; Burow and Käsbohrer, 2017). Reservoirs of resistance have emerged due to the irrational use of antibiotics in humans, animals, communities, and associated environments, resulting in the persistence of drug residues or resistance genes in the environment. Multiple environmental reservoirs are part of ABR dissemination, including soil, water, hospital, industrial, farm waste, and various polluted ecological niches (Marti et al., 2014; Huijbers et al., 2015). The trafficking or spillover of pathogens with resistance genes is easier within or among humans, animals, and the associated environment (Woolhouse and Ward, 2013; Holmes et al., 2016). However, recently, some researchers have questioned the contribution of animal production to the ABR crisis as they described limited livestock or aquaculture-associated infections in humans (Chang et al., 2015).

It is plausible to address ABR by considering a multi-sectoral and coordinated One Health approach (O’Neill, 2015; Holmes et al., 2016). One Health endorses that sustained growth of the human population is affected by climate change and the reduction in natural resources so that various disciplines may work together for the global health security of humans, animals, and the ecosystem (So et al., 2015). In the context of ABR, human health is considered as a priority with interdependent animal and associated environmental health due to the emergence and increased prevalence of multidrug-resistant (MDR) superbugs such as *Staphylococcus aureus (S. aureus), Escherichia coli (E. coli)*, and *Klebsiella pneumoniae (K. pneumoniae)*. It has been estimated that ABR poses a significant health burden on the global population (Prestinaci et al., 2015; O'Neill, 2016), and an estimated 0.2 million neonatal deaths have been reported due to MDR pathogen-associated sepsis. Out of these neonatal deaths, about 0.1 million were reported from five countries, i.e., China, Congo, Nigeria, Pakistan, and India (Malik et al., 2019).

This review highlights the “One Health One World” perspective on ABR by presenting its interlinked and multi-sectoral nature with key health or disease considerations of humans, animals, and the associated environment. The challenges and factors linked with implementing the One Health approach and containment of ABR at a local and global level are highlighted. The key guidelines and One Health action plan initiated by the Tripartite’s Commitment Food and Agriculture Organization (FAO)-World Organization for Animal Health (OIE)-WHO to control zoonotic diseases and ABR are also summarized.

## Delineating the One Health Domains Involved in ABR

According to the WHO recommendations, the ABR should be specifically used to describe resistant bacteria because humans and animals themselves have not become antibiotic-resistant (Hernando-Amado et al., 2019). The ABR is a major component of One Health studies as it is a leading concern for global public health, food safety, and food security. The major domains of One Health directly linked with ABR include surveillance and reporting of ABR, tracking transmission dynamics of MDR pathogens at the human-animal-environment triad, awareness, community education, policy decisions, and preparation of the technical workforce to decrease ABR. In human clinical settings, “antibiotic-resistant infections or antibiotic-resistant patients’ may be used because this term describes the patient that harbors the antibiotic-resistant pathogen and who is a potential risk factor for the distribution of ABR. In an environmental setting, hospital-acquired infections associated with resistant pathogens may be described as “antibiotic-resistant hospitals” to restrain the dissemination of ABR (Donker et al., 2012). The term “antibiotic-resistant environments”, like contaminated soil, polluted rivers, sewage, waste, etc., would allow grading of different environmental niches with their potential risk of ABR transmission.

To develop a criterion for identifying ABR resistant patients, hospitals, and environments, the WHO has prioritized the detection of resistant bacteria harboring antibiotic resistance genes (ABRGs) by employing various novel molecular tools like real-time PCR, gene capture tools, and whole-genome sequencing, etc. (Lanza et al., 2018; Tacconelli et al., 2018; Pärnänen et al., 2019). A detailed analysis of ABRGs and resistant bacteria involved in disseminating ABR from the contaminated environment to the non-contaminated environmental niche is also vital (Martínez, 2011).

The molecular epidemiology and genetic relatedness of ABR at the human-animal-environment interface are critical One Health components for reducing the global burden of ABR microorganisms. Integrated-type of surveillance programs with regular evaluation are required for ABR to confirm their appropriateness and applicability in the relevant domain. In this regard, several tools describing different aspects of surveillance are available, e.g., Progressive Management Pathway (AMR-PMP; FAO), Network for Evaluation of One Health (NEOH; EU COST), and SURVTOOLS (FP7-EU) (Nielsen et al., 2020).

## Political Recognition of ABR as a One Health Concern

In 2014, the first notable transcript which highlighted that a One Health strategy is required to tackle the ABR was published by WHO in collaboration with FAO and OIE in a report describing the surveillance of microbial resistance (Badau, 2021). Afterward, this formal tripartite alliance improved worldwide coordination and encouraged multidisciplinary collaboration between public and animal health in combination with food safety. The FAO-OIE-WHO has declared the ABR as one of the prioritized concerns for mutual action. Later in 2016, Jim O’Neill report was placed on the agenda of the G7 and G20 international summits, where they confirmed that the problem of ABR should be solved through the One Health approach. In the same year, the United Nations dedicated their 71^st^ General Assembly 2016 meeting to the issue of ABR. The members of the house-made a resolution the denounces that inappropriate use of antibiotics in human health, animal health, agriculture & livestock, food, and aquaculture as the main cause of ABR. Additionally, that resolution designated the ABR as an urgent risk that would need to be controlled with extreme responsiveness across the globe (O'Neill, 2016; Badau, 2021).

## Antibiotics and Their Relationship With Various Pillars of One Health

The usage of antibiotics, persistence of antibiotic residues, and presence of resistant bacteria in the human-animal-environment niches are associated with the One Health triad due to the interdependence of these pillars in the food chain and environment. Some antibiotic groups are reserved mainly for humans’ use, such as the drugs used for therapeutic management of tuberculosis (e.g., isoniazid). Whereas some drugs, such as ionophores and flavophospholipol are specifically recommended in veterinary settings. Similarly, many antibiotic classes are regularly prescribed at human and animal clinics (McEwen and Fedorka-Cray, 2002; Van Boeckel et al., 2015). Furthermore, some antibiotics, such as streptomycin, tetracyclines, etc., are used in horticulture as prophylactic measures or to treat bacterial infections, such as fire blight of pears and apples (Vidaver, 2002).

In humans, antibiotics are mainly used to treat clinical infections and for prophylactic purposes, such as post-surgery cases. However, the application of antibiotics in veterinary settings is different between pets and food-producing animals. In pets, the prescription of antibiotics is generally comparable to those in humans (McEwen and Collignon, 2018). While it is used for treating clinical infections (Landers et al., 2012) or used as feed additives and growth promotors in food-producing animals **(** **Table 1** **)**. Antibiotics in poultry are often administered to the whole group through water or feed without any clinical indications for preventive purposes. These practices are common among flocks of broilers, layers, and pens of pigs (McEwen and Fedorka-Cray, 2002; McEwen and Collignon, 2018). The regular prophylactic application of antibiotics in humans is not very common and is generally used only to manage serious communicable infections such as meningococcal infections. However, in those cases, antibiotics are administered to individuals with close and prolonged contact with the infected person. For example, in the case of meningococcal infection in school children, prophylactic use will be limited to those students who belong to the same household and will not be administered to all students in a classroom or school (Theilen et al., 2008).

**Table 1:** The global impact of antibiotic treatment on food-producing animals.

Class	Trade name	Generic name	Livestock animals	Administration route	Purpose	Side effects	Ref
Penicillin	Pfizerpen	Benzylpenicillin (penicillin G)	Cattle, pigs, sheep, turkeys, horses. Dogs, cats, calves	SC, IM,	Increased food intake, weight gain, and improved herd Health. Pneumonia in cattle, sheep arthritis, sepsis in pigs, horses, sheep, cats, dogs	Vomiting and shivering, pain at the injection site	(Liu et al., 2015)
Sulfonamide	Sulquin Di-Methox Injection-40%, Sulfasol	Sulfaquinoxaline Sulfadimethoxine	Rabbits, dogs, poultry dogs, turkeys, cats	Oral, IV	Control liver coccidiosis, feed additive, growth promotion	Crystallization of sulfonamides can occur in the kidneys with high doses	(Liu et al., 2015)
Polypeptides	Baciferm, Vetropolycin	Bacitracin, Zinc, Bacitracin	Food-producing animals. Beef cattle, dairy cattle, poultry, and swine, turkey	Topical, IM	Increase the feed conversion ratio. Improved growth, meat production weight gain. Feed additive	Itching, burning, or inflammation	(Page and Gautier, 2012)
Aminoglycosides	Amifuse E Amiglyde-V, GentaVed 50, GentaVed 100 NeoMed 325	Amikacin Gentamicin Neomycin	Cattle and sheep, chickens, goats, lambs, piglets, horses, turkeys	IV, IM, Oral	Growth promotion, weight gain to cure mastitis	Dehydration, renal dysfunction, cardiac dysfunction, endotoxemia, renal necrosis	(Ziv et al., 1982; McGlinchey et al., 2008)
Amphenicols	Florum	Florfenicol	Poultry, birds	Oral	Shows activity against many chloramphenicol-resistant bacteria, growth promoter	Induces early embryonic death	(Al-Shahrani and Naidoo, 2015)
Tetracycline	Aureomycin, Terramycin	Chlortetracycline, Oxytetracycline, Doxycycline	Calves, lambs, poultry, and swine	IV, IM	Growth promoting	Nausea, anorexia, vomiting, and diarrhea	(Angelakis, 2017; Granados-Chinchilla and Rodríguez, 2017)
Cephalosporins	Naxcel Cobactan	Cephalosporins (ceftiofur), (Cefquinome)	Chicks, turkey, cattle, goats, pigs, sheeps	IM, SC	Growth promoter, selectively inhibit Firmicutes allow Bacteroides	Anorexia	(Chatfield et al., 1984; Hornish and Kotarski, 2002)
Polymyxins	Colistin sulfate, florfenicol	Amoxicare-Vet, Dafull	Food-producing animals. Beef cattle, dairy cattle, poultry, and swine	IV, IM	Increase the feed conversion ratio. Improved reproduction ability, promote growth	Risks of toxicity and neurological disorders	(Zhou et al., 2011)
Macrolides	Tylan 40, Tylan 100 Biaxin Erythro-200	Tylosin, clarithromycin, erythromycin	Poultry, broilers, cattle, pigs, lambs	Oral, IV	Antimicrobial feed additive. Improved performance, microbiome modification, lipid metabolism, and energy reaping	Can be fatal to pregnant animals	(Dibner and Richards, 2005; Lin et al., 2013; Plumb, 2018)
Streptogramins	Stafac.	Virginiamycin	cattle, pigs swine, turkey, and broiler chickens	Oral	Growth promotion, meat production weight gain. Feed additive	Increase resistance	(Dumonceaux et al., 2006; Page and Gautier, 2012)
Glycopeptides	Coxistac G, Sacox	Salinomycin	Poultry, broilers, turkeys, birds	Oral, IV	Growth promotion, control infection with coccidia microbiome modification, immune regulation. increased food intake, weight gain, and improved herd health	Leg weakness, diarrhea, and depression	(Zhou et al., 2011; Fung et al., 2013)
Lincosamides	Lincomix	Lincomycin	Swine	Oral	Modification of the small intestinal microbiota of swine permits more efficient intestinal and, therefore whole-animal growth	Transient diarrhea or loose stools	(Nielsen and Gyrd-Hansen, 1998)
Fluoroquinolones	Orbax Baytril	Orbifloxacin Enrofloxacin	Dogs and cat poultry	Oral	Health improvement, growth-promoting, used for better skin, soft tissues in pet animals. Improves feed efficiency, thereby increasing productivity	Diarrhea, and lack of appetite, cartilage, sometimes blindness in cats. Reducing the performance of incubated eggs and hatching chicks	(Gouvêa et al., 2015; Papich, 2015)
Monensin	Rumensin	Monovet 90	Cattle and goat	Oral	Increase feed efficiency and weight gain, increase milk production, and decrease milk fat	Adaptation of microbiota may occur; varies with diet and animal	(Dubuc et al., 2010)

Some experts justify prophylactic use of antibiotics in cases of infectious outbreaks detected on some farm or flocks of animals. The antibiotic administration is recommended when the risk of prophylactic bacterial infection is high due to mixing of new animals, crowded or unsanitary conditions, the stress of transport, and age-related factors (National Research Council Committee on Drug Use in Food a, 1999). In animals, the use of antibiotics as growth promoters is considered an important factor contributing to resistance due to administration at a sub-therapeutic level and for a prolonged time. These conditions favor the development and spread of drug-resistant microbes in animals and between groups of animals. These drug-resistant microbes may enter humans either through the environment or through the animal food chain (McEwen and Collignon, 2018).

**Figure 2:**
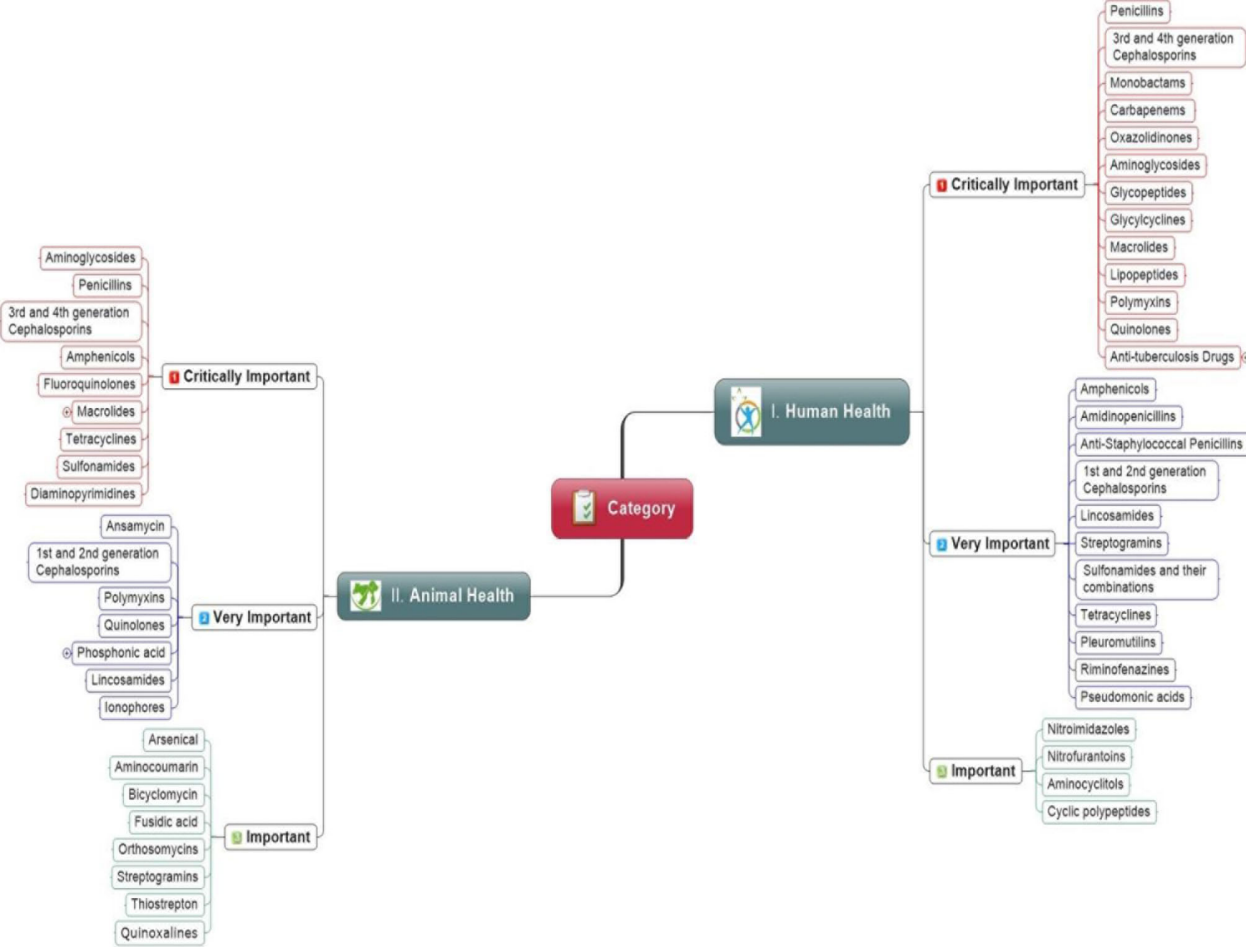
Priority wise classification of antibiotic classes for human and animal health recommend by the World Health Organization and World Organization for Animal Health.

The antibiotics dose regime in food animals generally lasts for more than two weeks. However, it often lasts for the whole production period, as seen in the chickens, i.e., 36 days. However, the imprudent use of antibiotics in food animals has been restricted because it may increase ABR risk in society (Prestinaci et al., 2015; Hoelzer et al., 2017; Aslam et al., 2018). Different studies have suggested that antibiotics may create 1 to 10% economic benefits when used as growth promoters in poultry production. These benefits are usually derived from the prophylaxis perspective of drugs instead of improving feed efficiency or production benefits. Therefore, a few large poultry production companies are now promoting their chicken or chicken products without antibiotic use at any stage. i.e., from the hatchery to the farms (Hao et al., 2014; Collignon and McEwen, 2019; Hedman et al., 2020). There are increasing concerns about the use of antibiotics as growth promoters to compensate for poor hygienic practices, improper housing, and the absence of proper animal health management (Singer et al., 2016; Kirchhelle, 2018). The FAO-OIE-WHO has recommended that antibiotics must not be used for growth promotion to tackle the growing burden of resistance. Keeping the importance of ABR issues in mind, the guidelines have been designed and implemented in Europe, Canada, and the United States (McEwen and Collignon, 2018). The OIE and WHO have categorized drugs into three classes, i.e., critically important, highly important, and important to animals and humans, as shown in **Figure 2**.

## Global Distribution of ABR *via* One Health

**Figure 3:**
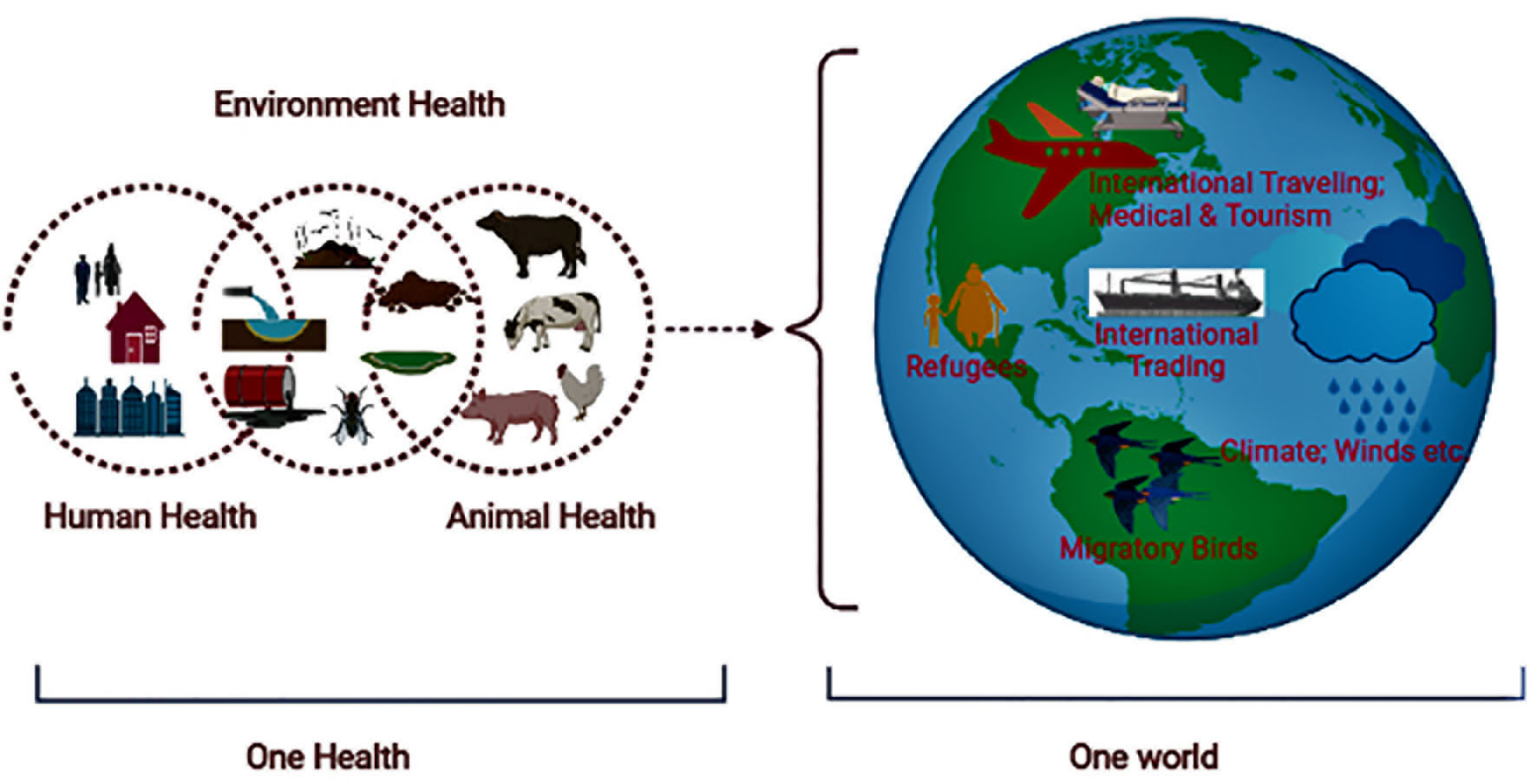
Illustration showing the ABR in a One Health One World perspective.

Although it is believed that ARGs existed in nature before the discovery of antibiotics, the emergence and spread of ABR in pathogenic strains occurred in response to the development and use of these agents. It is considered that the current menace of ABR developed gradually among pathogenic bacteria through evolution in response to various factors (Martínez, 2011; Hernando-Amado et al., 2019). The important One Health factors of global ABR distribution include intensive food production, globalization of food distribution, international travel (e.g., the spread of drug resistance genes), changing climate, increased population density or growth, and urbanization. The global burden of ABR is plausibly associated with excessive use of antibiotics in animals (food, pets, aquatic) and humans, antibiotics sold over the counter, increased international travel and trading, migratory birds, refugees, climate change, poor sanitation/hygiene, and the release of non-metabolized antibiotics or their residues into the environment (Iskandar et al., 2020) (**Figure 3**). These factors result in genetic selection pressure on bacteria (vertical and horizontal transmission of drug resistance genes between similar or different bacteria), ARG distribution in the environment, and the spread of MDR pathogens in the community (Cycoń et al., 2019).

Regardless of many national and international regulations for antibiotics use, a study has highlighted that global antibiotic consumption has significantly increased (between 2000 and 2015), and its consumption will double in 2030 (Klein et al., 2018). This increased consumption trend will be high in the low- and middle-income economies because of development projects and improvements in public access to health services. It is believed that the actual quantity of antibiotics being sold in low- and middle-income countries (LMICs) is underestimated because the antibiotics are usually sold in these countries without any medical prescription or sold over the counter, which favors the emergence of antibiotic-resistant bacteria (Yong Kim et al., 2005; Auta et al., 2019). It is also observed that the increased availability of antibiotics together with improved sanitation and vaccination programs have greatly contributed to minimizing endemic diseases and mortality in African countries (Keenan et al., 2018). It is crucial to reduce the global use of antibiotics to manage the growing burden of ABR, and antibiotics usage may be recommended according to geography or the burden of infectious diseases.

The extensive use of antibiotics is also reported in the animal health and agriculture sectors. According to estimates, two-thirds of total antibiotic consumption is used in animal production, but as growth promoters, their use in animal has been restricted in several countries (Aarestrup et al., 2001). According to estimates, the global use of antibiotics was 131,000 tons in animal production during 2013, which is projected to increase to 200,000 tons in 2030 (Van Boeckel et al., 2017). The increase in average individual meat consumption due to increased per capita income in the LMICs is the main reason for the increased demand for antibiotic use in animal production (Van Boeckel et al., 2015). The competition among countries regarding the production and export of meat and meat products has also favored the increased use of antibiotics in livestock production (Gray and Merchant, 2018). Although antibiotic consumption has increased during the past decade in the United States, the major proportion (around 80%) of antibiotics were purchased for use in fish farming and livestock production during 2011 (Van Boeckel et al., 2015; Done et al., 2015). Generally, previous waste treatment procedures used in the livestock sector are inadequate to eliminate resistant superbugs and ARGs, which may be disseminated through water and soil. Different contact routes of ARGs from the contaminated environment to humans include ingestion or inhalation of resistant superbugs, which may be attributed to the rise in multi-drug-resistant human infections. The etiology/reason for ARG dissemination and acquisition from animal waste to human gut microbiota has yet to be proved (Manaia, 2017). Detailed investigations are needed to explain the maintenance, persistence, and survival mechanisms of superbugs and ARGs at farm/waste levels and their transfer to humans. It will help to understand the spread of ARGs in new hosts and environments and the emergence of ABR at the environmental-human nexus (He et al., 2020).

The most abundant pollutants in developed and underdeveloped countries are heavy metals, which are also being used as feed additives in food animal production (Dowling et al., 2016). Heavy metals and many biocides can co-select for ABR, stimulate horizontal gene transfer, and can alter the antibiotics dynamics in a particular natural ecosystem. Environmental selection of ARGs is caused by antibiotic or metal residues which are frequently used in livestock, resulting in selection pressure; mobile genetic elements (MGEs) mediate horizontal transfer of ARGs. Therefore, it is important to explore the role of various metals and biocides in the selection and spread of antibiotic resistance *via* the environment (Fang et al., 2016; Jutkina et al., 2018; Hernando-Amado et al., 2019). Stringent measures are required to decrease the risk of metal-induced co-selection of ARGs, ABR, and maintenance of microbial communities in metal-rich environments of public health concern.

The increased economic activity in the LMICs is associated with the increased consumption of antibiotics. Consequently, the risk of developing ABR microbes has increased. The ABR pathogens excrete through human and animal stools can accumulate in the natural ecosystems and spread throughout the environment (Walsh et al., 2011; Karkman et al., 2019; Agramont et al., 2020). It is suggested that public health interventions should be implemented to improve water and food quality in the emerging economies and better sewage disposal.

Wastewater treatment plants, drinking water, and coastal water are reservoirs for disseminating ABR genes or microbes, and it is difficult to control their dissemination or risk management (Ma et al., 2017; Leonard et al., 2018). Different studies have shown that the pattern of ABR genes present in wastewater treatment plants is similar to clinical settings (Pärnänen et al., 2019). The genetic relatedness and metagenomics tools are used to predict and continuous surveillance of ABR microbes (Hendriksen et al., 2019). The guidelines for a threshold level of antibiotic-resistant bacteria and genes need to be defined to ensure a better quality of drinking water, the release of sewage water, and safer recycling of water for domestic and agricultural use (Moura et al., 2010; Yang et al., 2017).

The drug residues can cause water pollution upon their release into the sewage water; therefore, rapidly degradable antibiotics should be developed, along with better waste-water treatment techniques to decrease antibiotic residues in water systems, natural ecosystems, and environmental selection pressure (Chin et al., 2018; Rodríguez-Chueca et al., 2019). The on-site waste-water treatment plants are important since they can decrease the level of antibiotics, antibiotic-resistant bacteria, and genes in the downstream water systems (Paulus et al., 2019).

Natural or artificial alterations in the ecosystem can play an important role in spreading antibiotic resistance due to the interaction of humans, animals, vectors (flies, fleas, or birds), and the environment (Fuller et al., 2012; Beugnet and Chalvet-Monfray, 2013). Weather patterns and variations in the oceans can alter the distribution of bacterial pathogens, including antibiotic-resistant bacteria in various continents (Martinez-Urtaza et al., 2016). Pathogenic bacterial species and antibiotic resistance genes were observed during flooding and natural disasters (Yu et al., 2018). However, these phenomena need attention to explore their role in the dissemination of ABR and MDR pathogens.

## Antibiotic Resistance Mechanisms in Bacteria

Generally, there are two resistance mechanisms which a bacterium may use to resist the antibiotics (A) intrinsic mechanism of resistance (2) acquired mechanism of resistance. Resistance mechanism in which bacteria resist the action of the antibiotics through functional characteristics or in-built structural component is termed as intrinsic resistance. For example, *Pseudomonas* has the ability to resist a broad spectrum antibiotic named Triclosan, it displays this resistance due to the presence of an insensitive target (*fabI)* site for triclosan (Chuanchuen et al., 2003; Blair et al., 2015). Another example is related to daptomycin (Lipopeptide) that is unsuccessful against enterobactrales, because anionic phospholipids are present in lower proportion in the cytoplasmic membrane of Gram-negative bacteria. So, Ca2+-mediated insertion of daptomycin into the membrane is reduced (Miller et al., 2016). Moreover, some antibiotics are usually unable to go across the bacterial membrane, which is considered as intrinsic mechanism of resistance as well. For instance, vancomycin targets d-Ala-d- Ala peptides and impedes the peptidoglycan network in Gram-positive bacteria, whereas it cannot cross the outer membrane in Gram-negative bacteria (Kim et al., 2015). There are certain genes e.g. *AmpC*, *bla*_SHV,_ *TrxA* (thioredoxin), *TrxB* (thioredoxin reductase) etc. which are also considered as liable for intrinsic resistance against various antibioitcs like β-lactams, aminoglycosides, rifampicin, triclosan and flouroquinolones etc (Kakoullis et al., 2021).

In addition to intrinsic, bacteria may also exhibit the acquired resistance mechanism to restrain the action of antibioitcs. There are different mechanisms that help the pathogen to acquire the resistance such as inadequate penetration of the antibioitcs, drug efflux which results in decreased concentration of antibiotics inside the cell, target modification and antibiotic inactivation/hydrolysis (Blair et al., 2015).

At present, incidence of MDR pathogens harboring acquired resistance determinants is mounting across the globe. The reason behind this health concern is the production of different enzymes like extended spectrum β-lactamases (ESBLs), metallo-β-lactamases (MBLs), carbapenemases, which are associated with the resistance against cephalosporins and carbapenems. Well know ARGs such as *bla*_TEM_, *bla*_CTXM_, *bla*_KPC_, *bla_NDM_*, *bla*_VIM_, and mcr-1 are responsible for the emergence of MDR strains of E. coli, P. aeruginosa etc (Aslam et al., 2018). The *mcr-1* (plasmid mediated colistin-resistant) is a striking example of One Health dissemination of ARGs, as first isolation of mcr-1 was reported in China from raw meat (Liu et al., 2016). Moreover, From Pakistan, *E. coli* harboring mcr-1 was detected in specimens collected from migratory birds and human isolates (Lv et al., 2018).

## Estimation of ABR in a One Health Context

Human-animal-environment interfaces create chances for one or the other population to be a reservoir of ARG bacteria, which can be disseminated in any direction (Hassell et al., 2017). Two theoretical models have been presented which depict the ABR transmission route between these interfaces. One is the clonal transfer of resistant bacteria and the second one is horizontal gene transfer (HGT) (Chang et al., 2015). Each of these interfaces would have variable transmitting levels of resistance, which has been studied to comprehend the interface between resistant bacteria, host, environment, and MGEs (Hoelzer et al., 2017).

One of the promising tools currently used to estimate the level of ABR in these interfaces is WGS. It has been recognized in a study with large genomic data sets that revealed that most *E. coli* O157 outbreaks were related to the consumption of contaminated animal food, plant food products, and contaminated abattoir processing (Dallman et al., 2015; Butcher et al., 2016; Rowell et al., 2016). To understand the exact undertones of the problem, we have to improve our understanding of human-animal-environment interfaces by employing high-resolution genomics.

Conventional microbiological procedures like culturing bacterial pathogens or sequencing isolates need extra logistics and may not be practicable in the field. An alternative approach to address this problem a pathogen-independent strategy may be used, i.e., metagenome sequencing, which is good enough to detect whole genetic material in a sample (Bragg and Tyson, 2014; Lanza et al., 2018; Guitor et al., 2019). However, this tool may have less sensitivity as it depends upon the sample composition and sequencing procedure. The solution to this problem is to use a target-based sequencing procedure with specific probes for different ARGs (Lanza et al., 2018; Guitor et al., 2019). Metagenomics enables us to assess the pathogen variation and dissemination of ARGs between different niches of One Health. Additionally, innovative strategies like metagenome Hi-C provide insights into pathogen association with various ARGs (Pehrsson et al., 2016; Stalder et al., 2019). The WGS-based genetic relatedness among the isolates from various sample sources is helpful in understanding the possible transmission routes between these One Health niches. Lastly, an accurate metadata record is extremely crucial, and it should be done according to the harmonized procedures and metadata management guidelines (Griffiths et al., 2017).

## Maintenance and Dissemination of Drug-Resistant Bacterial Clones at the Human-Animal-Environment Interface

The emergence of methicillin-resistant *S. aureus* (MRSA) demonstrated a well-known evolutionary line by which ABR clones are disseminated globally (Lakhundi and Zhang, 2018). The emergence of resistant mutants results from continued exposure of susceptible bacteria to antibiotics and their dissemination at the human-animal-environment interface (Munita and Arias, 2016). The underlying mechanisms associated with ABR clonal expansion are progressive antibiotic exposure or a potentially a genetic background. These factors direct the fitness cost, such as chromosomal variations that enhance ABR plasmid carriage or compensatory mutations in the case of a rifampicin-resistant strain of *Mycobacterium tuberculosis* (Hughes and Andersson, 2017). Once established, the ABR clones may find new opportunities for broad geographical dissemination and spill-over into other host populations, depending on transmission modes and the degree of antibiotic selection they encounter (Ward et al., 2016; Baker et al., 2018).

Mobile genetic elements (MGEs) are considered to serve as a vehicle for the dissemination of resistance at the human-animal-environment interface. *K. pneumoniae* is considered a host of different mobile ABR genes and has played a significant role in disseminating different extended-spectrum β lactamases and Carbapenemases across the globe (Tzouvelekis et al., 2012). This link can be associated with the dissemination of MDR *K. pneumoniae* from and between different domains of One Health. Horizontal gene transfer in *K. pneumoniae* provides a channel for ABR gene trafficking from a massive genetic pool into small subpopulations of bacteria.

The presence of other resistance mechanisms, like fluoroquinolone resistance (Fuzi, 2016), is likely to be determined by extensive antibiotic use followed by selective pressure and MDR clonal expansion, e.g., *Enterobacteriaceae* (Davies and Davies, 2010; Prestinaci et al., 2015). It is evident in the evolution of a clonal strain of *Mycobacterium tuberculosis* through *katG* mutation that may proceed with further mutations resulting in isoniazid resistance (Jagielski et al., 2014).

ABR among animals can impact human well-being if human microbiomes and animal microbiomes share similar antibiotic-resistant genes (Marshall and Levy, 2011). It has been shown that the risk of cross-species ABR transmission is rare (Cheng et al., 2019). However, it is difficult to discriminate between pathogens arising from foods or from animal origins. In the context of “exclusive mutual” bacterial colonization of either animals or humans *via* adapted clones, the chance of ABR transmission (from animals to humans) seems connected to specific clones, acting as shuttles of ABR through infecting and colonizing both kinds of hosts (Delahoy et al., 2018). However, high-risk ABR clones are usually spread through food (animal-human contact) and *via* farm animal diversity. For example, the *S.aureus* CC97 strain has jumped from livestock to humans. Likewise, human-animal spread has also been reported, such as the MRSA CC398 lineage jumping into livestock due to high selection pressure (Aires-de-Sousa, 2017).

Some studies have revealed limited relatedness of MDR bacterial pathogens at the human-animal interface. A cross-sectional study in East England (Ludden et al., 2019) compared the core genome of *E. coli* isolated from livestock farms and retail meat. Overall, 41 different resistance genes were detected in different proportions in the isolates from livestock. Significantly sharedgenes include ESBLs, *sul1, sul2, strA, strB, tetA, and tetB.* It was observed that *E. coli* associated with human infections does not originate from livestock sources directly as they found genetically distinct isolates from livestock and human. While it has also been observed that the same animal species from different animals have significant microbiome relatedness. It has been reported that *E. coli* phylogroup B2 is significantly (68%) associated with human infection as compared to animal infection (1%) (Nash et al., 2010). The ubiquitous distribution of various ARGs at the human-livestock interface may be linked with transmission *via* MGEs as described previously for ESBL genes (de Been et al., 2014). A similar study was reported from East England last year (Ludden et al., 2019), which showed no evidence for livestock as a dissemination source of MDR *K. pneumoniae* to humans. However, the study results revealed that the hospital environment is a significant source of MDR *K. pneumoniae* associated with severe human infections and pan-genome analysis of the isolates showed significant genetic diversity in *K. pneumoniae*. Another previous study has reported that *K. pneumoniae* has higher transmissibility between different domains of One Health as compared to *E. coli* (Gurieva et al., 2018).

The Vancomycin-resistant *Enterococcus faecium* (VREfm) is listed among the global priority list of antibiotic-resistant bacterial pathogens by the WHO (WHO, 2017). Previously, livestock was suggested as a possible disseminating source of VREfm or vancomycin resistance genes to humans, which was related to vancomycin (Avoparcin), used as a growth promoter in Europe. Later on, that drug was banned in 1997 in Europe. A decade ago, the increased incidence of VREfm among broilers was reported ST10, ST13, and ST370. Contrary to this fact, a study conducted in the recent past has reported limited strain sharing of VREfm among livestock and humans from the UK (Gouliouris et al., 2018). Genetic relatedness was observed by comparing livestock isolates and human isolates associated with bloodstream infections. No VREfm was detected in livestock in that study. Overall, 26 different resistance genes were identified in human and livestock isolates, while the depth analysis revealed limited sharing of ARGs between livestock and humans. Additionally, a limited overlap was observed between the isolates of hospital and livestock origin (Gouliouris et al., 2018).

*Salmonella typhimurium (S. typhimurium)* and *Campylobacter* contributed a lot to acquiring antibiotic resistance, leading to food safety and lower livestock production issues. The *S. Typhimurium* strain 104 became MDR with the acquisition of a 43kb genome, showing high resistance against five of the first-line antibiotics (Leekitcharoenphon et al., 2016). The presence of MGEs among various types of bacteria also seems to be a leading determinant of ARG’s between different bacterial clones or species and between different hosts, such as the plasmid coding for ESBL (plasmid IncI2) carries the MCR-1 gene, which is responsible for encoding resistance to Beta-lactamase and colistin. Similarly, MDR plasmid p60006 can be found in *Enterobacteriaceae* shuttle clones, and the Inc18 plasmid caries the vanA gene in enterococci species (Partridge et al., 2018; Klemm et al., 2018). The chain of infection in the human-animal-environment can be broken by preventing the dissemination of drug-resistant bacterial clones, MGEs, and ARGs through the One Health approach.

## Risks of ABR for Public, Animal, and Environmental Health

The ABR lowers the efficacy of antibiotics at clinics and leads to an increased incidence of infection and severity (O’Neill, 2018). the extensive use of antibiotics in animals significantly contributes to ABR among human microbes, primarily, enteric microbes like *E. coli*, Campylobacter spp., *Enterococcus* spp., and *Salmonella* spp (O’Neill, 2015). The exposure of pathogens to biocides like antiseptics, disinfectants, and heavy metals both in environmental niches and in animals may co-select for ABR (Wales and Davies, 2015). Among foodborne pathogens, non-typhoidal *Salmonella* is a well-known pathogen associated with gastroenteritis in humans and is responsible for about 94 million cases, including 155000 deaths every year (Organization WH, 2014a). This ABR pathogen is generally spread by transporting animals, contaminating poultry and animal meat products through carrier animal feces (Organization WH, 2004). Fluoroquinolone and cephalosporin-resistant *Salmonella* is a leading public health problem in the world (Dutil et al., 2010). Therefore, therapeutic options for different groups, such as pregnant women and children, are limited due to drug toxicity issues. Cephalosporins are used to treat very serious infections. Further, it has been reported that fluoroquinolones used against *Salmonella* infections in food animals are a leading cause of quinolone resistance development (CVMP E, 2006). The surveillance data of the WHO revealed a low fluoroquinolone resistance rate among non-typhoidal Salmonella in the European region (2-3%), a wide range in Americas (0-96%), and higher rate in the Eastern Mediterranean (up to 40-50%) (Organization WH, 2014a). ABR continues to emerge in *Salmonella* strains and has been related to certain other life-threatening infections in humans (Helms et al., 2004).

Among different water and food-borne infections, *Campylobacter* infection is usually regarded as a self-limiting infection. Fluoroquinolone-resistant *Campylobacter* severe infection has also been reported due to prolonged antibiotic use (Helms et al., 2005). Fluoroquinolones are usually given as a mass antibiotic through drinking water in animals. In countries like Australia, this type of antibiotic has never been approved for use in poultry. Therefore, quinolone resistance against *Campylobacter* in Australia is low (Cheng et al., 2012). Similarly, macrolides are usually given as growth promoters, but resistance to macrolides has also been reported in *Campylobacter* (FDA U, 2013). In animals, *E.coli* causes many infections, such as enteritis, salpingitis, omphalitis, septicemia, synovitis, mastitis, and cellulitis (Mellata, 2013). Some bacterial strains are considered as gut commensals of humans and animals, while others behave as donors resistance genetic elements and opportunistic pathogens (Collignon, 2015).

The increasing incidence of *E.coli* infections in humans and animals is a serious health concern and is well documented in developing countries due to contaminated food and drinking water (Graham et al., 2014). Travelers may also acquire MDR *E. coli* from inadequate food/water or other people. ESBL producing *E.coli* is also well-documented as a source of infection in humans in developed and developing countries (Lazarus et al., 2015). By recognizing the One Health approach, the FAO-OIE-WHO alliance is trying to create an integrated surveillance system for food-borne ABR bacteria, to accurately calculate public health risks (Organization WH, 2017). The WHO has estimated the prevalence of *E. coli* resistance to third-generation antibiotics for the American population (48%) and Southeast Asia and Africa (70%). The health burden from 3^rd^ generation fluoroquinolone and cephalosporin-resistant *E.coli* infections revealed a 2-fold increase in 30-day and all-cause mortality (Organization WH, 2014b).

MRSA may cause different infections, such as bloodstream, wound, and skin in hospital and community settings (Lakhundi and Zhang, 2018). According to WHO estimates, the MRSA prevalence is 60% in Europe, 80-100% in Africa, and up to 90% in America. Health burden analysis of MRSA revealed a significant increase in all-cause, intensive care, and bacterium attributable mortality in patients with healthcare-related MRSA infections (Organization WH, 2014b). However, mortality rates for community-acquired MRSA bacteremia are generally reported to be lower than for sensitive strains of *S. aureus*. The MRSA strains that are pathogenic for humans have been expressed in many different animal species, and their spread to humans is mainly thought to occur *via* carrier animal contact. The major leading factors in MRSA transmission in animals are the use of antibiotics in food, livestock, international trade of animals, and lapses in biosecurity within/or between farms (Mehndiratta and Bhalla, 2014; Davis et al., 2017).

In the environmental health context, the ABR is associated with transmitting MDR pathogens and ARGs of public health concern (Banerji et al., 2019). Most drug resistance genes, pathogenic microbes, and antibiotics have environmental origins, such as soil and water (Berkner et al., 2014). Resistance to most drugs has already been demonstrated in bacteria during the pre-antibiotic time, but important evidence suggests that the activities of humans have a great impact on the development of “global resistome” (Gaze et al., 2013). Since antibiotics are produced in massive amounts annually, environmental niches play an important role in the persistence and spread of ABR microbes (Kraemer et al., 2019). Inadequate sewage and pharmaceutical waste treatment results in the release of many antibiotics into the water, which serves as a major source for the transmission of resistance genes/or resistant bacteria (Davies and Davies, 2010; Kraemer et al., 2019). Poor sanitation and international travelers, globalized trade in food as well as in animals serve as sources of global dissemination of resistance. In this regard, possible measures to address drug resistance include risk assessment, environmental monitoring, and proper control measures to reduce pollution from agricultural, industrial, and residential sources (Pruden et al., 2013).

## ABR and One Health in Lowand Middle-Income Countries

The global emergence and re-emergence of MDR bacteria or superbugs pose a serious threat to public health (disease burden, mortality, economic losses) in developing and LMICs. The dissemination and spillover of MDR pathogens is a consequence of excessive antibiotic use in animals and humans due to non-metabolized antibiotics or their residues in the environment (water, soil). The MDR pathogens persist across the animal, human, and environmental triangle or niche, and there is interlinked sharing of the animal-human-environment interface. In LMICs, animals and animal origin foods act as a reservoir of MDR pathogens due to misuse of antibiotics in veterinary practice as prophylaxis or growth promoters (Cleaveland et al., 2017). Resistant microbes are consistently present in our food chains, and they are shared between animals and humans directly and indirectly through our environment. The occupational risk of transmission of these MDR bacteria is very high for veterinarians, slaughterhouse workers, hatchery retailers, or handlers. Therefore, ABR is a trans-sectoral problem necessitating a trans-disciplinary, coordinated, and collaborative “One Health Approach” to tackle the public health issue. Since human and animal health, food/feed and animal production systems, and agro-ecological environments are directly associated with ABR. Therefore, a multidimensional One Health approach is direly needed to circumvent ABR globally (Gitaka et al., 2020). In this regard, the FAO-OIE-WHO alliance took the initiative in collaboration with public and private organizations to mitigate the global menace of ABR at the animal-human-environment nexus. The FAO-OIE-WHO action plan is based upon evidence (eco-epidemiology, integrated surveillance, and reporting), communication of the masses, good practices (biosecurity, agro-ecology, and the alternative to antibiotics), and legal framework (policy and codex Alimentarius) through the implementation of the One Health or food chain approach. A global initiative named the Global Antibiotic Resistance Partnership (GARP) was launched in 2009 to form an action plan for the control of ABR in LMICs. Under the Tripartite’s Commitment FAO-OIE-WHO initiative, regional, local, and global efforts have been launched to report ABR and implement action plans by different countries (Bank, 2010; Gitaka et al., 2020).

## One Health and Economics

The distribution of ABR in various regions of the globe is influenced by economic development and technology (Ruppé and Chappuis, 2017; Ruppé et al., 2019). The World Economic Forum can play a pivotal role in decreasing the burden of global ABR through infrastructure, regulations, human resource development, and public health interventions. From an economic perspective, the One Health approach can coordinate, communicate, and collaborate with various sectors, stakeholders, and policy makers to improve the interlinked health of humans, animals, and the environment. Policymakers take different corrective measures, including travel and tourism regulations, food and agriculture regulations, public health interventions, and monitoring and surveillance strategies to control ABR (Prestinaci et al., 2015).

It is challenging to shift from the current health paradigm to the One Health model based on diverse ecologies and geographies. To establish the One Health paradigm, a strong and considerable assessment of net benefits is essential. As a discipline, economics has two significant features to help us think about One Health: efficient resource usage and the marginal value of approach substitution. A substantial comparison of marginal benefits against marginal costs is necessary to transform the conventional health approach into a holistic One Health approach. For instance, a reduction in the disease burden is a potential outcome of the One Health paradigm. It may be determined in practical terms, like the decreased incidence/prevalence rate of the disease and further interpreted using different economic standard methods like a contingent valuation.

## One Health Scheme to Cope With ABR

The WHO has launched a Global Action Plan based on the One Health approach to fight against ABR, and it demands all the members around the world to follow the same guidelines while preparing their national action plans (McEwen and Collignon, 2018). Improved awareness and understanding of ABR is necessary and can be done through effective communication, education, and training. Every stakeholder of One Health should understand the One Health domains of ABR, including clinicians, veterinarians, farmers, industrialists, and policymakers. These components could minimize antibiotic usage in humans and animals/farms and contain ABR dissemination through the environment (McEwen and Collignon, 2018). The recognition, appraisal, awareness, and advocacy of ABR-related One Health dimensions include seminars by public and animal health organizations, local and global outreach programs for farmers, curriculum development, and linkage between various stakeholders of One Health (McEwen and Collignon, 2018; Manyi-Loh et al., 2018).

Evidence-based research and surveillance are essential to enhance awareness of the One Health dimension of ABR because they recognize the problems and drivers of ABR and plans for the control of ABR dissemination (Aarestrup et al., 2008; Perry and Wright, 2014; O’Neill, 2018). At present, specified and targeted research is indispensable to determine the drug resistance mechanisms, estimate the incidence rate of ABR in various ecologies, find cost-effective and appropriate alternatives to antibiotics, and support antibiotic stewardship (Laxminarayan et al., 2013; Huijbers et al., 2015; O’Neill, 2015; O’Neill, 2018).

**Figure 4:**
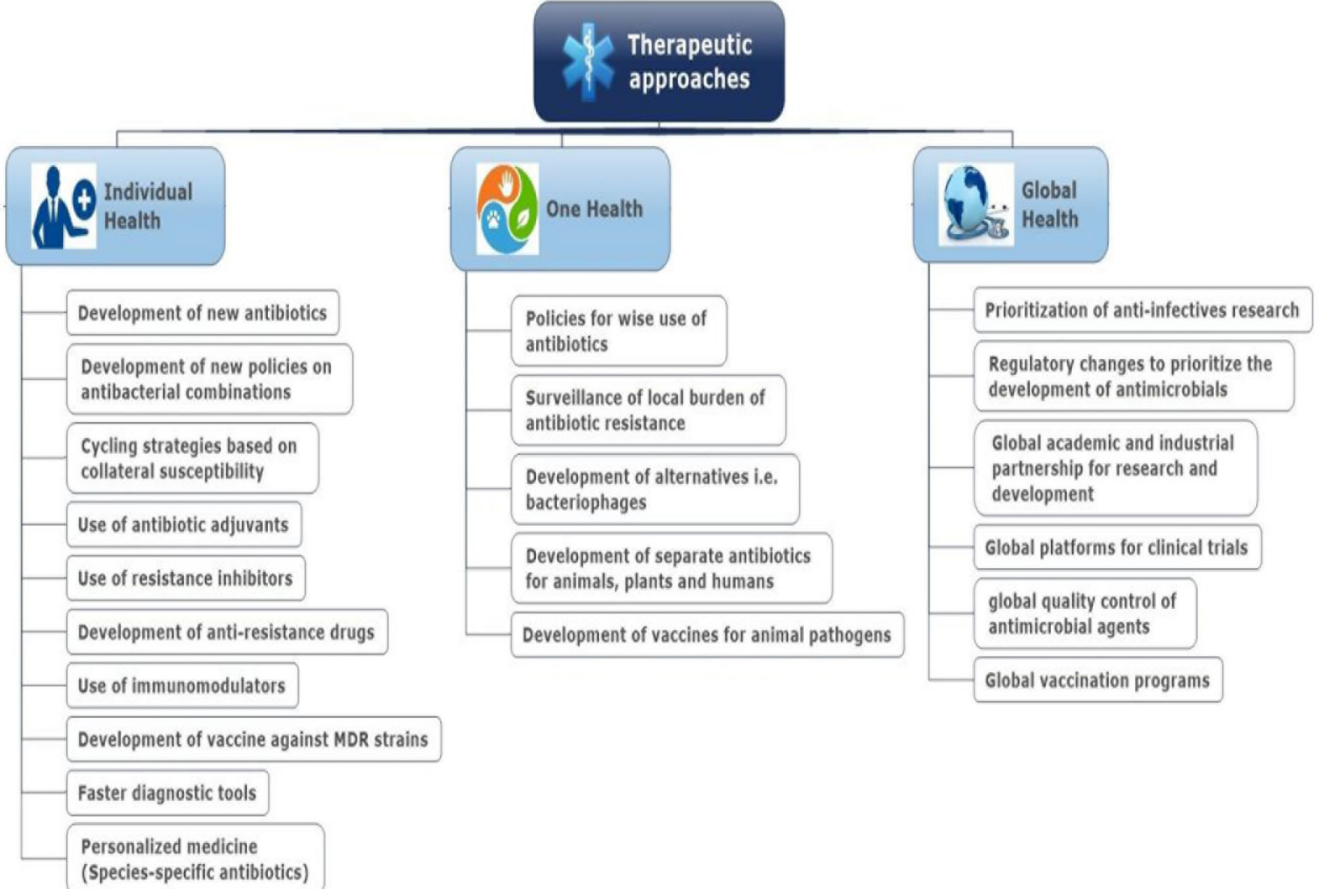
Therapeutic approaches to reduce the burden of antibiotic resistance.

Adequate sanitation and hygiene are crucial for infection control in healthcare settings, especially in hospitals, reducing the dissemination of ABR or ARGs from hospitals into the environment (Collignon, 2015). In the case of veterinary and farm settings, in addition to biosecurity, prudent use of antibiotics as therapeutics **(** **Figure 4** **)** or prophylaxis needs to be followed (Murphy et al., 2010). Dissemination of resistant pathogens and ARGs from the environment to humans may be reduced by implementing control measures to improve food and water quality, especially in LMICs (Gaze et al., 2013; Collignon, 2015; Singer et al., 2016).

For antibiotic usage, monitoring of antibiotic consumption, and various mechanisms for persistent upgrading of antibiotic utilization may be introduced to decrease ABR (Aarestrup et al., 2008). In this context, the European Union has banned antibiotics as a growth promoter in food-producing animals. The USA has banned extra-label use of 3^rd^ generation cephalosporins and fluoroquinolones in animals, and many countries around the globe ensure the use of antibiotics in animals with veterinarian prescriptions (Aarestrup et al., 2008; Scott et al., 2019). Antibiotic stewardship plans should be directed to ensure that antibiotics are used only for therapeutic purposes in health care settings for both humans and animals (Landers et al., 2012; Scott et al., 2019). The world forum may play its role in promoting and advocating the One Health approach to cope with global ABR.

## Alternatives to Antibiotics-One Health Perspective

Antibiotics are the central players in controlling bacterial pathogens, but decreasing their usage is vital because of the emergence of resistant pathogens and transmissible ARGs. The latest research findings enabled us to understand the gene exchange and pathogen interactions in various One Health niches, which is important for developing alternative antibiotic approaches (Aslam et al., 2018).

### Microbiota Modulation

Microbiota modulation through probiotics, fecal transplantation, or improved nutrition has been studied extensively in human and animal health contexts. Further, the dynamics of host-microbe interactions need to be improved through advanced genomics assays. Genomic tools may be very helpful in detecting the mechanisms and reservoirs of ABR in various niches of One Health. Additionally, these may also be useful for developing and testing novel interventions to be made to reduce the dissemination of ABR and ARGs. Specifically, microbial taxonomy and the presence of genes encoding for resistance, virulence, and toxin production can be determined through metagenomic sequencing. It also identifies various MGEs that may be transferred between various niches (Young, 2016; Brugman et al., 2018; Relman and Lipsitch, 2018).

### Gene Editing Techniques

Gene editing and transgenic techniques unwrap more options to control resistant bacterial pathogens (Proudfoot et al., 2019). It has been used to control bacteria responsible for mastitis, which requires significant antibiotic therapy, e.g., engineered lysozyme and lysostaphin (Oliver et al., 2005). Though these methods are still under regulatory procedures, such methods can provide a suitable alternative to antibiotics (Waltz, 2017). WGS-based mutagenesis to recognize the viral genes which influence replication is used to find novel targets for gene-editing (Han et al., 2018; McCormick et al., 2018).

### Vaccines

Vaccines also play a crucial role in controlling ABR because they reduce the need for antibiotic therapy (Lipsitch and Siber, 2016). Advanced genomic tools like functional and comparative genomics would be helpful in vaccine development. These tools may predict the conserved pathogen factors accessible to antibodies through secretion signals, which may be tested as a subunit vaccine, known as reverse vaccinology. This has been used to develop vaccines against mastitis, brucellosis, and *E. coli* infections. Moreover, transcriptional analysis of the pathogen inside the host may also be very helpful in selecting the targets. Recent advancement in this regard is transposon sequencing, which is useful in assigning phenotypes to variants. Although genomics is currently applied to discover antigens as vaccine candidates, genomic-wide host response investigations relevant to specific vaccines are required for comprehensive understanding (Seib et al., 2012).

### AV Inhibitors

Genome-based tools like RNAseq could find out the regulons, regulators, and various virulence factors required for the onset of infection and endurance of the pathogen inside the host. A promising alternative approach to disarming the pathogen using AV (anti-virulence) may also be used in which the infection’s expression of virulence genes required for the infection can be controlled. For example, a potential AV inhibitor known as virstatin, impedes gene expression in *V. cholerae* through ToxT (regulator) downregulation (Hung et al., 2005).

### Bacteriophages

Bacteriophages are the natural enemies of bacteria and destroy them during their life cycle. The emergence of ABR shifted the research interest towards the use of phages and engineered phage proteins like endolysins and depolymerases, etc. (Jault et al., 2019; Aslam et al., 2021; Patel et al., 2021),. Bacteriophages may be used in animal health systems to improve the health status of food-producing animals. It may also be used to decrease bacterial contamination during food processing and packaging. Bacteriophages may be used in aquaculture and the veterinary health system to control *E. coli* O157: H7, especially in food-producing animals (Sabouri et al., 2017; Almeida et al., 2019). Some issues related to bacteriophage therapy, such as safety and stability, need to be addressed before their use in health care settings. Another concern related to bacteriophages is that they may introduce virulence or ARG alleles into the bacteria, so they should be sequenced before use. Recently, bioinformatics tools have been developed to solve this problem, e.g., PHANOTATE, GeneMarks, Glimmers, etc. (Tzouvelekis et al., 2012),. It can specifically analyze the genome of bacteriophages for various factors by using available databases (Cui et al., 2017).

## Recent Advancements

The ABR is recognized as an expanding and dynamic health concern globally11, thus specifying the significance of advanced tools to delineate its undercurrents and to discover the diversity in detail. Novel computational and sequencing tools like Whole-genome sequencing (WGS) or Next-generation sequencing (NGS) have enhanced the applications of advanced tools for studies of ABR in different domains of One Health (Van Camp et al., 2020).

### Available Databases

In this regard, several databases are available online, like ResFinder, AMRFinder, CARD, and ARG-ANNOT, etc., a few databases that work for a single pathogen are also available, such as Kleborate for Klebsiella and MUBII-TB-DB or Dream TB for Mycobacterium tuberculosis (Flandrois et al., 2014; McDermott and Davis, 2021). Recently, a summary describing different free online resources for ARG identification in bacterial pathogens has been published (Hendriksen et al., 2019). Moreover, NCBI has published a database that has about 5000 resistance genes identified from across the globe.

### Metagenomics

At present, a sequence-based approach to studying genomes from a mixed microbial community known as metagenomics is being applied to understand and decipher the complexity of ABR in different niches of One health sector. Generally, bacteria take on HGT to acquire ARGs. So it is important to decipher the HGTs, which may offer a comprehensive understanding of ABR dissemination in and among different One Health niches. Since metagenomics is a high-throughput DNA sequencing tool, it made the ARG analysis achievable and practicable. It is a suitable tool which permits the right to use the genome data available in environment without culturing the specimens for the isolation of bacteria (de Abreu et al., 2020).

Metagenomics helps to delineate the microbial diversity by finding the genes and through reconstruction of complete genomes of microbial community (Chen and Pachter, 2005; De, 2019). One of the benefits this tool possesses is its sensitivity, because it detects species abundance and identifies ARGs of microbial community. Presently, metagenomics is considered as an alternative tool to rRNA sequencing and has been used to study microbial diversity in clinical and environmental specimens (Escobar-Zepeda et al., 2018).

Over the last decade, a few sequence-based tools like shotgun metagenomics, amplicon sequencing, and functional genomics have been used to study ABR. Functional genomics is used extensively to detect and identify ARG variants and novel ARGs (Mendes et al., 2017; Collignon and McEwen, 2019; de Abreu et al., 2020). The SmartChip real-time PCR system (Takara, Japan) is a high-throughput tool used frequently to study ABR, especially in environmental sources (Franklin et al., 2021).

On the other hand, there are few challenges which disturb the efficiency of metagenomic analysis. Firstly, less sensitivity towards microbial population present in minority which may also harbor ARGs (Lynch and Neufeld, 2015). Secondly, lower specificity to detect the bacterial variants that may have significant impact, because variants may develop diverse phenotypic traits (Forslund et al., 2013). These shortcomings may be resolved by combining the metagenomics sequencing with functional genomics (Chistoserdovai, 2010; Lam et al., 2015).

### WGS

On the other hand, at the bacterial level, WGS is considered a powerful tool to study ABR and predict the strains’ resistance profile, but the inconsistency between culture-based testing and WGS must improve. Classification of ARGs and different sources of One Health is crucial because of the complexity of microbial communities present in different domains. In this regard, some recent investigations by employing these techniques have been conducted to categorize specific ARGs that may pose health threats, revealing that MGEs are associated with the dissemination of ABR among different sectors of One Health (Su et al., 2019; Mackenzie and Jeggo, 2019). In coming times, these advanced tools will be vital for understanding the dissemination and transmission of ABR and ARGs in different domains of One-Health.

### Diagnostics

Prompt and precise diagnosis has a significant impact on antibiotic susceptibility and consumption. Currently, novel techniques have been developed that allow a quick pathogen identification process and its susceptibility to various antibiotics. Various tools which may be helpful in this regard include rapid immunochromatography, automated time-lapse microscopy, and Matrix-assisted Laser Desorption Ionization (time of fight)-Mass Spectrometry (MALDI-TOF MS) (Lau et al., 2014; Ghosh et al., 2015; Singhal et al., 2015; Florio et al., 2020). However, there are some limitations to using these techniques, like running costs, as the reagents used in these tools are highly expensive, especially for researchers from developing countries. Additionally, these tools require a considerable number of qualified and trained personnel. A few molecular biological techniques are also available as diagnostic tools to identify ARGs, e.g., Xpert Carba-R and FilmArray BC-ID (Maurer et al., 2017; Baer et al., 2021). The PCR/electrospray ionization-mass spectrometry platform is a substantial advancement in the field; it may detect over 500 pathogens and various ARGs in a few hours (Wolk et al., 2012; Kailasa et al., 2019). The main advantage of these tools is that they may be executed on samples, directly reducing the time for pathogen detection or susceptibility testing. According to the available literature, such methods will be routine laboratory procedures in the coming future.

## Research Gaps

It is now well understood that different ecological niches play a crucial role in the evolution and dissemination of ABR and ARGs among various One Health domains. Given that, stakeholders and scientists are looking for further explanations regarding the drivers and mechanisms involved in ABR distribution across the globe. Additionally, they seek to estimate the risk burden and to identify appropriate interventions. In 2017, renowned scientists from various parts of the world gathered in a workshop held in Sweden, arranged by the University of Gothenburg Center for Antibiotic Resistance Research and the Swedish Research Council (SRC), where they defined the main research gaps in the One Health scenario to tackle ABR.

They categorized four imperative research directions, which include; a) quantified contribution of various sources of ABR and ARGs to the environment; b) evolution of resistance-related with human-environment interface; c). Impact of resistant environmental pathogens on human and animal health; d) the practicability and efficiency of various economic, technological, behavioral, and social interventions to fight ABR. Delineating all these four research domains is vital to comprehending the mounting concerns related to ABR. For that reason, we should encourage funding agencies, policy makers, and all stakeholders to help researchers do multidisciplinary research, which would be very effective in bridging the knowledge and research gaps in this field.

## Challenges and the Way Forward

Many countries across the globe have made their own national action plan to cope with ABR based on the One Health approach. It has been established that One Health is essential to the fight against ABR (White and Hughes, 2019). It fosters linkage among different domains/sectors, which operated separately in the past, and plays a potential role in better coordination among various sectors. Some countries are practicing integrated surveillance systems for antibiotic usage and ABR. Implementing regulations regarding the use of antibiotics in veterinary practice as growth promoters and the role of industries in restricting the use of antibiotics in food-producing animals has been initiated. Despite these advancements, reported data revealed that antibiotic usage both in humans and animals is still on the rise across the globe, and resistant pathogens are re-emerging (Van Boeckel et al., 2015; O’Neill, 2018). Significant measures and implementation of regulations are required to address global human, animal, and environmental health security.

There are many challenges to improving antibiotic stewardship in One Health, like motivation inadequacy, limited awareness, and malpractices in antibiotic usage, and inadequate regulatory or policy measures in various countries (Aarestrup et al., 2008; O’Neill, 2018). The developing world is slow in adopting scientific advancement and accepting the evidence of public and animal health impacts posed by excessive antibiotic use in food-producing animals (Bengtsson and Greko, 2014). Advanced molecular techniques like whole genome sequencing, metagenomics, metadata analysis, and phylogenetic studies are indispensable for a better understanding of global ABR. These advanced techniques will support us in understanding the transmission dynamics of resistant superbugs and ARGs among humans, animals, insects, plants, water, and soil (Oniciuc et al., 2018; Kraemer et al., 2019; Wee et al., 2020). One Health is a subject that promotes health *via* interdisciplinary collaboration by collecting data across various domains of One Health to explain the route of ABR transmission at the human-animal-environment interface. In the future, microbiome analysis of various domains of One Health will be vast and challenging. Antibiotic stewardship plans must be practical towards the reduction of antibiotic use. Additional antibiotic stewardship obstacles such as over-the-counter availability of antibiotics, particularly in LMICs, lack of authentic data on antibiotic usage, and inadequate therapeutic guidelines in different countries need to be addressed (Edwards et al., 2018; Larsson et al., 2018).

## Conclusion

The global emergence and spread of ABR necessitates promoting a coordinated and multidisciplinary One Health approach to reduce human, animal, and environmental health risks. The global spread of ABR pathogens and ARGs due to misuse of antibiotics, inadequate sanitation facilities, and insufficient control measures negatively impacts global public health. The global assessment of the One Health approach and the FAO-OIE-WHO commitment would help ABR prevention through awareness programs, education about antibiotic usage, advocacy with political commitment, and antimicrobial stewardship.

## Author Contributions

ZB, BA and XX designed the study and finalize the article. MK, MR, MA NY, TS, SM, TC, AS and MHR search the available literature and wrote initial version.

## Funding

This work was supported by the key research and development programs (2019ZF004 and 202103AC100001) of Yunnan Province.

## Conflict of Interest

The authors declare that the research was conducted in the absence of any commercial or financial relationships that could be construed as a potential conflict of interest.

## Publisher’s Note

All claims expressed in this article are solely those of the authors and do not necessarily represent those of their affiliated organizations, or those of the publisher, the editors and the reviewers. Any product that may be evaluated in this article, or claim that may be made by its manufacturer, is not guaranteed or endorsed by the publisher.
