# Corrigendum: Antibiotic resistance: One Health One World outlook

**DOI:** 10.3389/fcimb.2024.1488430

**Published:** 2024-09-25

**Authors:** Bilal Aslam, Mohsin Khurshid, Muhammad Imran Arshad, Saima Muzammil, Maria Rasool, Nafeesa Yasmeen, Taif Shah, Tamoor Hamid Chaudhry, Muhammad Hidayat Rasool, Aqsa Shahid, Xia Xueshan, Zulqarnain Baloch

**Affiliations:** ^1^ Department of Microbiology, Government College University Faisalabad, Faisalabad, Pakistan; ^2^ Institute of Microbiology, University of Agriculture, Faisalabad, Pakistan; ^3^ College of Veterinary Medicine, South China Agricultural University, Guangzhou, China; ^4^ Faculty of Life Science and Technology, Kunming University Science and Technology, Kunming, Yunnan, China; ^5^ Public Health Laboratories Division, National Institute of Health, Islamabad, Pakistan; ^6^ Faculty of Rehabilitation and Allied Health Sciences, Riphah International University, Faisalabad, Pakistan

**Keywords:** One Health, antibiotic resistance, human, animal, environment

In the published article, there was an error in affiliation 4. Instead of “Faculty of Life Science and Technology, Kunming University of Life Science and Technology, Kunming, Yunnan, China”, it should be “Faculty of Life Science and Technology, Kunming University Science and Technology, Kunming, Yunnan, China.”

The authors apologize for this error and state that this does not change the scientific conclusions of the article in any way. The original article has been updated.

